# The pathogenesis of bone metastasis in solid tumors: a review

**DOI:** 10.3325/cmj.2021.62.270

**Published:** 2021-06

**Authors:** Ivan Vičić, Borislav Belev

**Affiliations:** 1 Department of Oncology, University Hospital Center Zagreb, Zagreb, Croatia; 2 University of Zagreb School of Medicine, Zagreb, Croatia

## Abstract

Owing to its frequent occurrence and severe clinical picture, bone metastasis is an important problem in the clinical course of tumor diseases. Bone metastasis develops when the physiological remodeling process is disrupted by tumor cells via the same molecular mechanisms used by native bone cells. The process includes molecular crosstalk between osteocytes and osteoblasts and osteoclasts. Osteolytic bone metastasis, most often seen in breast cancer, is characterized by promoted differentiation and function of osteoclasts and reduced osteoblast function. Tumor cells take advantage of factors released by bone tissue resorption, thus establishing a vicious cycle that promotes the metastatic process. In osteoblastic metastasis, most often seen in prostate cancer, osteoblast function and differentiation are promoted, while osteoclast activity is reduced, resulting in net gain of bone tissue. Mechanisms involved in the early stages of bone metastasis and cancer cell dormancy have been understudied, and their exploration may pave the way for potential therapeutic strategies. Tumor affects the bone marrow microenvironment via exosomes, soluble factors, and membrane-bound ligands. In this way, an initial lesion is established, which after a variable duration of disseminated tumor cells dormancy progresses to an overt condition. The current review deals with basic mechanisms involved in bone metastasis formation and propagation. We illustrated a disparity between the diversity and number of factors included in the disease pathophysiology and the number of available and developing therapeutic options. We also examined new therapeutic strategies affecting molecular pathways.

The bone is the third most frequent metastasis site, behind the lungs and liver (1). Given common clinical manifestation and a high degree of related disability, bone metastases pose a serious problem in the clinical course of tumor diseases. Since bone metastases present frequently in tumor diseases, they have an important predictive role. Namely, the median survival from the diagnosis of bone metastases is 12-53 months for prostate cancer, 19-25 months for breast cancer, 48 months for thyroid cancer, 6-7 months for lung cancer, 6 months for melanoma, 6-9 months for bladder cancer, and 12 months for kidney cancer (2).

All known mechanisms taking part in bone metastasis process are related to the disorders of physiological bone remodeling. Although the traditional division of bone metastases into osteolytic and osteoblastic is still widely accepted, these categories are increasingly viewed as only extremes of a continuum (3).

## Tumor cell migration, adhesion, and invasion

Cancer cells begin to infiltrate a distant site by migrating from the circulating blood through the blood vessel wall to the extracellular space of the bone. Here, we described the role of molecules that mediate cancer cells homing in the bone extracellular matrix and enable cancer cell adhesion to the matrix molecules and other cells.

Blood vessels in the bone marrow (sinusoidal blood vessels) are fenestrated and lack the usual supporting structure of the capillaries. This specific structure increases the likelihood of tumor cells extravasation through the vessel wall (4).

After cancer cells cross the vessel wall, a stable cell mass is established in the new environment through mechanical adhesion. An important adhesion molecule and a potent chemotactic factor for various stages of hematopoietic cells is CXCL12, also known as stromal-derived factor 1 (5). Its effects are mediated by CXCR4 and CXCR7 receptors on tumor cells. The interaction alters the ratio of cytoskeletal elements in terms of polymerization and polarization of actin, pseudopodia formation, and enhancement of adhesion to epithelial cells and extracellular matrix elements (6). Similar chemotactic properties to the CXCL12/CXCR4 action are also exhibited by CXCL16 and its receptor CXCR6 (7).

Annexin II is a 36-kDa membrane protein and an extracellular matrix component. In osteoblasts, it participates in the adhesion of hematopoietic stem cells and regulation of hematopoietic stem cell survival within the stem cell niches. The interaction of annexin II and its receptor contributes to the tropism of tumor cells (8).

Another factor playing an important role in tumor cell adhesion is the interaction of E- and N-cadherin in heterotypic adherence junctions (9). Breast cancer cells expressing E-cadherin produce more bone metastases compared with other metastatic foci (10).

Mechanical interactions of tumor cells with the extracellular matrix are mediated by integrins. Integrin α_2_β_1_, which binds collagen type I, has been observed in prostate cancer cells that produce bone metastases (an effect reversed by the action of a specific antibody), but not in the cells that produce visceral metastases (11). In breast cancer cells, α_v_β_3_ and α_v_β_5_ integrins have been found to mediate the adhesion to bone extracellular matrix proteins such as sialoprotein, vitronectin, and osteopontin (12).

Among factors participating in tumor diseases pathogenesis are members of small leucine-rich proteoglycans, a family of matricellular proteins: decorin, biglycan, asporin, and lumican. A lower decorin concentration was found in stromal cancer tissue and the extracellular matrix of metastases than in normal bone tissue (13). In tumor cells, decorin inhibits tyrosine kinase receptors, such as epidermal growth factor receptor, type I insulin-like growth factor receptor, and hepatocyte growth factor receptor (or mesenchymal-epithelial transition factor) (14,15).

Another group of matricellular proteins are small integrin-binding ligand N-linked glycoproteins, five of which take part in bone tissue function and various stages of metastasis process (13). Osteopontin anchors osteoclasts to the bone matrix by binding to integrin α_V_β_3_ (16). Osteopontin gene polymorphisms correlate with different bone metastasis formation potential, and an induced osteopontin expression in breast cancer cells increases the bone metastasis formation potential (17,18).

An important factor in bone metabolism and metastasis are cellular communication network (CCN) matricellular proteins (13). An increased expression of CCN2 (also known as connective tissue growth factor [CTGF]) protein has been identified in bone metastases of breast cancer compared with normal breast epithelial cells and other metastatic foci (18,19).

## Disseminated cancer cell dormancy and early phases of metastatic process

Circulating tumor cells detected in peripheral blood samples are considered a poor prognostic factor (20). Disseminated tumor cells are those that have overcome all obstacles from the primary tumor site to the target organ and have not yet established their activity at the distant site. A subset of disseminated tumor cells are cancer stem cells, characterized by low mitotic activity, resistance to chemotherapeutics, but also by a starting cell clone at the distant site (21).

The local tissue microenvironment that maintains and regulates the activity of stem or progenitor cells is called a niche. The bone marrow contains the perivascular and endosteal niche. The perivascular niche is located near the bone marrow sinusoids, and is populated by a sinusoidal endothelium, pericytes, dividing hematopoietic stem cells, bone marrow stromal cells, reticular cells (CAR cells), and others. The endosteal niche is located near the surface of the mineralized bone matrix and is populated by bone marrow stromal cells, osteoclasts, various developmental stages of osteoblasts, and quiescent hematopoietic stem cells (22).

An important part of the bone microenvironment are osteocytes, the most abundant bone cells. They attract tumor cells to the bone tissue by secreting CXCL12, an already mentioned chemotactic and adhesion molecule (23). Osteocytes affect tumor cells directly via several mechanisms. They downregulate Snail, a factor involved in epithelial-to-mesenchymal transition, thus favoring epithelial traits and tumor cell colonization of the bone (24). Osteocytes that inhibit osteoblast activity secrete dickkopf 1 (DKK1) and sclerostin, factors involved in bone turnover regulation, which in the context of the bone metastasis favors bone degradation (25). A direct contact of osteocytes and tumor cells establishes Notch signaling, a process inducing osteocytes apoptosis and enhancing tumor cell proliferation (26). Since osteocytes are mechanosensitive cells, their interplay with tumor cells is affected by mechanical stimuli. Physiological mechanical stress activates connexin 43 (a hemichannel) on osteocytes. This leads to the release of adenosine triphosphate (ATP), which inhibits tumor cell proliferation, whereas its metabolites stimulate metastatic cells. Therefore, the net effect is determined by the balance between ATP and its metabolites. The ATP amount released depends on the mechanical stimulation strength (27,28).

A variety of roles in bone metastasis formation is played by adipocytokines, factors secreted by bone adipocytes. Adipocytokines CXCL12, ANGPTL2, and ANGPTL4 increase vascular permeability and act as a chemoattractant for tumor cells (29,30). Proinflammatory cytokines IL-1beta, IL-6, TNF-alpha, and CXCL1 and CXCL2 induce myeloid-derived suppressor cells, which inhibit innate and adaptive immune response (31,32), while leptin promotes CSC properties and enhances the metastatic potential (33). Adipokines participate in the induction of osteomimicry (an expression profile of the tumor cells similar to that of the native bone cells, mostly osteoblasts) mediated by the Runx2 transcription factor (34). Bone marrow adipocytes can alter the metabolism of metastatic cells. Prostate cancer cells cultured with bone marrow adipocytes had increased levels of lipid-transfer proteins FABP4, CD36, and perilipin 2 (35).

Tumor cell dormancy refers to the G0/G1 phase of the cell cycle (36). This quiescent state triggers the development of metastatic disease many years after the primary tumor development. It also enables tumor cells to adapt to or resist chemotherapeutics and protects the cells from immune system recognition. The induction of the tumor cell dormancy can be explained by several theories, the most convincing one being that bone marrow microenvironment modulates tumor cell activity (37).

**Figure 1:**
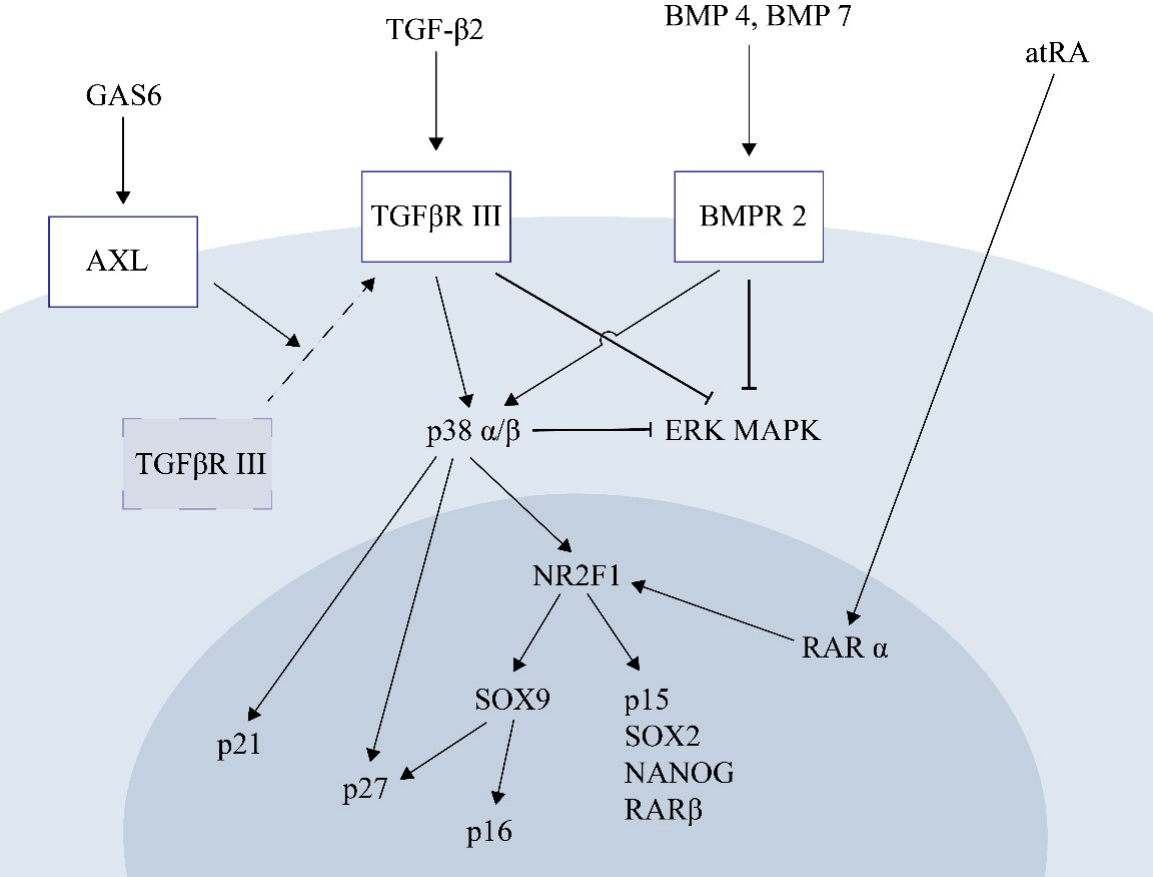
Cancer cell dormancy induction. Abbreviations: GAS6 – growth arrest-specific protein 6; BMP – bone morphogenetic protein; TGF – transforming growth factor; ERK MAPK – extracellular signal-regulated kinase mitogen-activated protein kinase; atRA – all-trans retinoic acid; RAR – retinoic acid receptor; NR2F1 – nuclear receptor subfamily 2 group F member 1; SOX – SRY-related HMG-box.

Dormant cells are characterized by the predominance of p38 MAPK signaling pathway activity and the inhibition of ERK MAPK pathway, with *vice versa* being true for active tumor cells (38,39). Another proven dormancy trigger is MKK4, an upstream factor of p38 in the MAPK signaling pathway (40). In addition to p38 action, an important dormancy regulator is NR2F1, a nuclear hormone receptor and transcriptional regulator that activates NANOG, SOX2, SOX9, and RARβ transcription factors. Tumor cell dormancy is further mediated by p15, p16, and p27 inhibitors of cyclin-dependent kinases. The effect of NR2F1 appears to encompass epigenetic mechanisms and a reduced Myc oncogene activity (Figure 1) (37,41).

After extravasation, tumor cells of the mesenchymal traits colonize the perivascular and the endosteal niche. These niches are rich in factors that regulate hematopoietic stem cell behavior. One of these factors is growth arrest-specific protein 6 (GAS6), secreted by osteoblasts in the endosteal niche. The annexin II-mediated link between tumor cells and osteoblasts promotes the expression of tyrosine kinase receptors AXL, Sky, and Mer on tumor cells, whose ligand is GAS6. Thus, the GAS6/AXL signaling is established and tumor cell dormancy is initiated (42,43). The p38 MAPK signaling pathway is also stimulated by TGF-β2, trans-retinoic acid, BMP4, and BMP7, which are secreted in the endosteal niche (39,44). Another dormancy-inducing factor in tumor cells is thrombospondin 1, secreted by the endothelium. On the other hand, the proliferating endothelium secretes TGF-β1 and periostin, factors that activate tumor cells. Thus, cells located in the perivascular niche receive signals that direct them to either dormancy or metabolic and mitotic activity (45).

Tumor cell dormancy is interrupted by resorptive activity, ie, the release of growth factors by the osteoclast activity. The interaction of vascular cell adhesion protein (VCAM-1) and integrin α_4_β_1_ on osteoclast precursors promotes osteoclastogenesis. VCAM-1 expression is initiated by the action of NFκB, which is stimulated by the action of receptor activator of nuclear factor kappa-B ligand (RANKL), PTH(rP), or IL-6 (46). Growth factors are released from the bone matrix by enzymes such as ADAMTS1 and MMP1, and MMP7 stabilizes RANKL (47,48). Tumor cells also secrete heparanase, which promotes osteoclast activity (49). Osteoclastic activity can be potentiated by hypoxia-induced lysyl oxidase expression independently of the enzyme's usual activity on the extracellular matrix collagen fibers (50).

In breast cancer, bone metastases have been associated with the presence of miR-10a and miR-10b (microRNAs), although their target mRNAs have not been accurately identified. These miRNAs are upregulated by the transcription factor RUNX2, while miR-10b is upregulated by the transcription factor TWIST1. Thus, in the early phases of metastatic process miRNAs are part of regulatory mechanisms with known participants, ie, RUNX2 and TWIST1 (51,52). Furthermore, miR-135 and miR-203 expression was associated with a decreased RUNX2 expression in metastatic breast cancer, making them a potential therapeutic target (53). Other miRNAs associated with the suppression of these processes are miR-33a targeting PTHrP (mRNA) (54) and miR-335 targeting RANKL (55).

In addition to miRNAs actions within tumor cells, miRNAs transfer from tumor to non-tumor cells via exosomes has been described (56). Exosomes secreted from breast cancer tumor cells fuse with endothelial cells, importing miR-105, which reduces the expression of ZO-1 protein, an important component of the tight junctions (57). miR-122 is translocated by exosomes from metastases cells to niche cells, reducing the amount of M2 pyruvate kinase, decreasing GLUT1 transporter expression, and thus facilitating the metabolic supremacy of tumor cells (58).

## Osteolytic bone disease

In physiological conditions, bone formation and bone resorption are in a precisely regulated dynamic equilibrium, also known as the process of bone remodeling. Bone metastases in various tumor diseases, such as kidney cancer, non-small-cell lung cancer, malignant melanoma, thyroid carcinoma, non-Hodgkin's lymphoma, multiple myeloma, and breast cancer, are characterized by the predomination of resorptive processes (59). The major cause of bone tissue resorption is an increased osteoclast activity. However, tumor cells also reduce osteoblast activity by secreting a group of factors and by harnessing bone tissue mechanisms and substances for their progress, thus establishing a positive feedback system (3).

In bone remodeling process, the communication between osteoblasts and osteoclasts is mediated by Ephrin (Eph) B2 and EphB4 membrane receptors. EphB4 is found on osteoblasts and bone marrow stromal cells, and EphB2 on osteoclasts (60). This interaction reduces osteoclast activity and promotes osteoblast action. In bone metastases, the presence of tumor cells in the bone reduces the interaction of these receptors, ie, decreases the contact between the bone tissue cells (61).

An important role in the humoral stimulation of osteoclast function by tumor cells is played by RANKL. Its activity is mediated by the receptor activator of the nuclear factor κB (RANK), which is located on osteoclast precursors (3). RANKL binding to RANK triggers an intracellular cascade that involves the binding and activation of multiple TNF receptor-associated factors and downstream activation of numerous intracellular signaling pathways: nuclear factor κ-Β (NFκB), nuclear factor of activated T-cells c1, c-Jun, and melanogenesis associated transcription factor. Their action triggers the transcription of effectors important for the osteoclast action: αvβ3 integrin, cathepsin K, calcitonin receptor, and TRAP. These effectors promote bone resorption, and some have become therapeutic targets (62). RANKL function is affected by osteoprotegerin, a member of the tumor necrosis factor receptor superfamily secreted by osteoblasts and bone marrow stromal cells, which binds RANKL to impair its interaction with RANK. Thus, the resorption extent is determined by the osteoprotegerin to RANKL ratio (3).

RANKL secretion is increased by PTHrP. The NH_2_-terminal portion of PTHrP is very similar to that of parathyroid hormone (PTH), so PTHrP action is mediated by PTH receptor (PTHR1) (63). PTHrP secretion has been observed in over 90% of metastatic breast cancer cells characterized by osteolytic bone metastases (3). The RANKL/RANK system is also affected by tumor-secreted IL-11, which increases the RANKL level while decreasing the osteoprotegerin and PTHrP levels (64).

Differentiation of osteoclast precursors into osteoclasts, which increases bone tissue resorption, is promoted by many other interleukins, including IL-1, IL-6, IL-8, and IL-18 (65). IL-3 acts both through the RANKL/RANK system and directly on osteoclast precursors. Osteoclastogenesis is promoted also by macrophage inflammatory protein 1α. This protein acts as a chemotactic factor for osteoclast precursors and induces osteoclast differentiation by a RANKL-independent mechanism (61,66). Cyclooxygenase type 2 expression in osteoblasts is induced through MAP kinase activity, and consequently an increased PGE 2 concentration acts in an autocrine manner (mediated by EP4 receptor) to enhance RANKL production and osteoclast differentiation (67). TNF-α secreted by tumor cells and bone marrow stromal cells has a dual role of promoting osteoclast differentiation and inhibiting osteoblast function (61).

An important factor in the development of bone metastases is DKK1, an inhibitor of the Wnt/β-catenin signaling pathway. This protein plays a double role in tumor metastasis at different sites: it inhibits breast cancer lung metastasis by modulating the noncanonical Wnt signaling pathways formation and stimulates bone metastasis formation by modulating the canonical Wnt signaling pathways (68). DKK1 is increasingly secreted in osteolytic bone metastases, especially in multiple myeloma, reducing the expression of RUNX2, a key transcription factor in osteoblast differentiation. In addition to its effect on osteoblast differentiation, it also stimulates osteoclast activity by reducing osteoprotegerin expression and by enhancing RANKL expression (69). Osteoblast function is also inhibited by sclerostin and sFRP2, two inhibitors of the Wnt signaling pathways (53,61), as well as by IL-7 and TNF-α (70).

**Figure 2:**
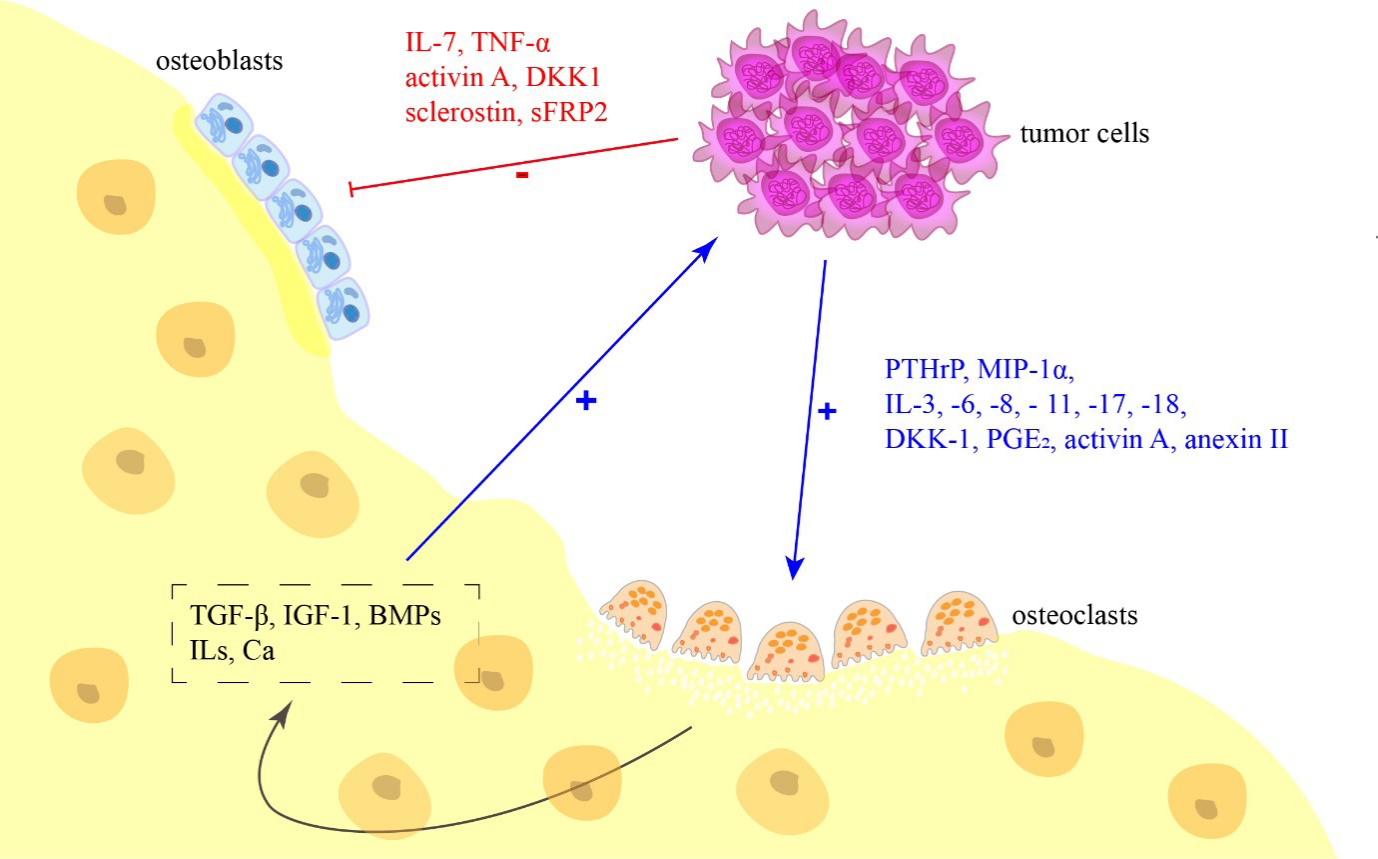
The vicious cycle concept: tumor cells stimulate osteoclast function and inhibit osteoblast function. The products of bone resorption act stimulatory on tumor cells. Abbreviations: IL – interleukin; TNF – tumor necrosis alpha; TGF – transforming growth factor; IGF – insulin-like growth factor; BMP – bone morphogenetic protein; PGE – prostaglandin E; DKK1 – Dickkopf-related protein 1; sFRP2 – secreted frizzled-related protein 2; PTHrP – parathyroid hormone-related protein; MIP-1α – macrophage inflammatory protein 1α.

Tumor cells exploit the communication pathways between osteoblasts and osteoclasts, which serve as qualitative, quantitative, and temporal regulators of bone remodeling. Bone resorption promoted by tumor cells (secretion of PTHrP, IL-11, CTGF, CXCR4, MMP-1) releases factors that stimulate tumor cells themselves, establishing a positive feedback mechanism (vicious cycle) that results in the spread of bone metastatic disease (Figure 2) (3,71,72). In addition to the already mentioned matricellular proteins, extracellular matrix contains numerous growth factors and cytokines. Of these, TGF-β, IGF-1, several types of BMPs, INF-γ, and several types of ILs are considered to stimulate tumor cells growth. Osteoclasts, more specifically low pH and secreted enzymes (MMP, cathepsin), release and activate bone matrix TGF-β. This cytokine stimulates tumor cells, acts chemotactically on mesenchymal stromal cells, and induces osteoblast differentiation, but in later stages inhibits osteoblast activity. It stimulates osteoclasts directly and indirectly via osteoblasts (73). In addition to growth factors, tumor cells respond to calcium ions (via CaSR) released in the process of bone resorption (74).

## Osteoblastic bone disease

Osteoblastic metastases, primarily found in prostate cancer, but also in small-cell lung cancer, Hodgkin's lymphoma, and medulloblastoma, have not been studied as extensively as osteolytic metastases (59). Although in this type of metastases bone mass is increased due to increased bone formation and decreased bone resorption, the functional structure and integrity of the bone is impaired (61).

Tumor cells secrete a number of factors that increase the osteoblast count and activity. Platelet-derived growth factor, a dimeric peptide (A and B unit), induces osteoblast differentiation and activity in bone metastases of prostate cancer (BB form) (75). Although the mechanisms are not fully elucidated, osteoblast activity is also enhanced by fibroblast growth factors and vascular endothelial growth factor (76,77). The action of insulin-like growth factor (IGF I and II) alone is insufficient to stimulate osteoblasts, so more aggressive tumors exhibit an increased IGF level and a decreased insulin-like growth factor binding protein level (78). Bone morphogenetic proteins (BMPs) secreted by tumor cells, especially BMPs 6, 7, and 4, stimulate bone formation. In addition to their effect on osteoblasts, they also affect angiogenesis (77). Prostate cancer has been shown to express Wnt 3a, 7b, and 10b, which modulate the canonical Wnt signaling pathways, playing a role in osteoblast differentiation and proliferation. Early prostate cancer has been demonstrated to express a Wnt inhibitor DKK1 as well. Therefore, the Wnt pathway signaling is determined by a balance of stimulatory and inhibitory signals (79). Another factor involved in the stimulation of osteoblast activity is endothelin 1 (ET1), which is secreted by tumor cells (80).

Prostate cancer bone metastases are also characterized by PTHrP secretion (72). This paradox may be most convincingly explained by the structural similarity of the NH_2_-terminal end of PTHrP to ET1 and by the binding of PTHrP to endothelin receptors after modification of the protein by secreted enzymes (eg, PSA) (81). Prostate cancer cells secrete urokinase plasminogen activator (uPA) and prostate-specific antigen (PSA). uPA is secreted by tumor cells in the form of high molecular weight-uPA (HMW-uPA). HMW-uPA is broken down into low molecular weight-uPA and the amino-terminal end, which binds to uPAR on osteoblasts and enhances their activity. uPA also acts on the inactive form of TGF-β, which is synthesized by osteoblasts. It also enhances IGF-I activity by breaking down IBFBP (82). Similarly to uPA, PSA (a serine protease) modifies the NH_2_-terminal end of PTHrP and participates in the release of active forms of growth factors (83).

In the early development of metastatic disease in the bone, tumor cells secrete substances that stimulate osteoblast activity. Osteoblasts form new bone tissue, which could soon physically limit the development of tumor cells. However, this is not a self-limiting process. The increasing osteoblast activity also increases the activity of osteoclasts (RANKL/RANK system, CCL2, IL-6), while bone matrix degradation leads to a release of growth factors. Tumor cells promote osteoblast activity as well as osteoclast activity, but the net result is bone tissue formation. RANKL secreted by osteoblasts acts on prostate cancer tumor cells via RANK. BMPs secreted from osteoblasts also stimulate tumor cells themselves (84). Therefore, a positive feedback mechanism is established through the factors released by osteoclast action. but also through factors secreted by osteoblasts.

## Therapeutic and diagnostic possibilities

Although pathogenetic molecular mechanisms of bone metastasis are becoming increasingly understood, our knowledge still has limited therapeutic application in terms of symptoms alleviation and slowing down the disease progression. Only a few agents are being accepted and confirmed as beneficial (Table 1), and they exploit a small number of mechanisms, leaving many potential strategies unused.

**Table 1:** Molecular mechanisms involved in different phases of bone metastasis and registered and non-registered therapeutic options*

		Registered and potential therapeutic agents	Reference
Migration, adhesion and invasion	CXCL12/CXCR4 and 7		(5,6)
	CXCL16/CXCR6		(7)
	annexin II/annexin II receptor		(8)
	E- and N-cadherin		(9)
	integrins α_2_β_1_, α_v_β_3_ and α_v_β_5_/ECM proteins		(11,12)
	decorin/tyrosine kinase receptors		(14,15)
	CCN2		(18,19)
Regulation of disseminated cancer cell dormancy and early metastasis	MKK4		(27)
	NR2F1		(37,38)
	GAS6/AXL	Cabozantinib (multi-targeted tyrosine kinase inhibitor)	(42,43,98)
	TGFβ2/TGFβR III		(39,44)
	BMP4,BMP7/BMPR2		(39,44)
	thrombospondin 1, periostin		(45)
	miR-10a, b (and other miRNAs)		(51,52)
	osteoclasts metabolism	bisphosphonates	(90)
Osteolytic bone disease	ephrinB2/ephrinB4		(60)
	PTHrP	PTH	(63)
	RANKL/RANK	Denosumab, everolimus (mediated by mTOR inhibition)	(3,91,93)
	DKK1	DKK1 specific antibodies	(68)
	sclerostin, sFRP2	Sclerostin specific antibodies	(53,61)
	cathepsin K	dutacatib, odanacatib, balicatib	(94)
	c-Src	bosutinib, dasatinib, ponatinib, vandetanib	(95)
Osteoblastic bone disease	PDGF		(95)
	BMP 6,7,4		(78)
	Wnt 3a, 7b, 10b		(79)
	endothelin 1	ET 1 antagonists	(80)
	uPA, PSA		(82,83)

*Abbreviations: CXCL – chemokine (C-X-C motif) ligand ;CXCR – C-X-C motif chemokine receptors; ECM – extracellular matrix; CCN2 – cellular communication network; MKK4 – mitogen-activated protein kinase kinase 4; NR2F1 – nuclear receptor subfamily 2 group F member 1; GAS6/AXL – growth arrest-specific protein 6; TGF – transforming growth factor; BMP – bone morphogenetic protein; RANKL – receptor activator of nuclear factor κ-Β ligand; DKK1 – Dickkopf-related protein 1; sFRP2 – secreted frizzled-related protein 2; PDGF – platelet derived growth factor; Wnt – wingless-related integration site; uPA – urokinase-type plasminogen activator; PSA – prostate specific antigen.

The first problem is a timely diagnosis. The cornerstone of clinical practice are still the traditional techniques such as computed tomography, bone radiography, bone scintigraphy, positron emission tomography-computed tomography, image-guided biopsy, magnetic resonance imaging. However, some new techniques are being perfected. The concept of nanoparticles as contrast agents is being widely accepted, eg, superparamagnetic iron oxide nanoparticles are used as a contrast agent in MRI (85). In addition, gold nanoparticles have been developed that bind specifically to prostate-specific membrane antigen, thus enhancing computed tomography-based prostate cancer metastasis diagnostics (86). Another approach is the use of biomarkers. The N-terminal cross-linked telopeptide of type I collagen and C-terminal cross-linked telopeptide of type I collagen, released by bone resorption, have been evaluated as breast cancer bone metastasis biomarkers (87). miR-214 can be used as a biomarker of breast cancer bone metastasis, and annexin A1 as a biomarker of small cell lung cancer bone metastasis (88,89).

A very common therapeutic strategy is the use of bisphosphonates, analogs of pyrophosphates, with high affinity for binding to calcium, ie, hydroxyapatite, during bone remodeling. These agents mainly inhibit the metabolism and apoptosis of osteoclasts (90). Another strategy is to disrupt RANKL-RANK interaction. IgG2 antibody denosumab inhibits osteoclastogenesis and osteoclast activity and impairs bone tissue resorption. In breast and prostate cancer bone metastases, denosumab has been shown to be more effective than zoledronate in delaying the first clinical manifestations of tumor disease, while being non-inferior in other tumors (91). Additionally, only bisphosphonates have been proven to reduce breast cancer metastasis to the bone (92).

Given that the action of RANKL, M-CSF, and TNF-α is mediated by mTOR, mTOR inhibitors have been classified as therapeutic strategies for bone metastases. mTOR inhibitors (everolimus) increase osteoprotegerin expression, osteoclast apoptosis, and probably promote osteoblast differentiation (93). Although cathepsin K inhibitors dutacatib, odanacatib, and balicatib have been shown to effectively inhibit bone resorption, their poor safety profile (odanacatib) led to the discontinuation of some studies; yet the therapeutic goal still exists (94). The proto-oncogene tyrosine kinase has also been identified as a therapeutic target, due to its role in osteoclast differentiation. Tyrosine kinase inhibitors bosutinib, dasatinib, ponatinib, and vandetanib have an inhibiting effect on bone metastases (95). Another possible therapeutic target are sclerostin-specific antibodies. Sclerostin, secreted by numerous tumor cells, inhibits osteoblasts and acts as a canonical Wnt signaling pathways inhibitor (96). Antibodies specific for DKK1, another Wnt inhibitor, are also under investigation (97). MET inhibitor cabozantinib (also inhibits VEGFR2, AXL, KIT, RET) has been studied as an inhibitor of angiogenesis and tumor growth in many tumor diseases. It also acts on bone remodeling, favoring osteoblastic activity (98).

Besides conventional chemotherapy (targeting both primary and metastatic foci) and targeted therapy, radiation has also been traditionally used. External beam radiotherapy is a bone metastasis treatment proven to be effective in reducing symptoms as well as decreasing tumor cell burden in the bones (99). A more focused technique is stereotactic body radiotherapy, which delivers a precise dose to a precise area, limiting damage to the surrounding tissue (100). Another form of delivering radiation is radionuclide-based therapy. Strontium-89 chloride and samarium-153-labeled ethylene diamine tetramethylene phosphonate are used to treat bone metastasis-related pain (101), while radium-223 chloride, an alpha-emitting radionuclide, is used to treat prostate cancer bone metastasis (102).

## Conclusion

Bone metastatic disease develops as a result of various mechanisms. Current knowledge on these mechanisms far exceeds the available successful therapeutic strategies. This fact indicates a lack of understanding of these processes, leading to inappropriate therapeutic options. A particularly pressing issue is the problem of cancer stem cells, ie, an incomplete understanding of the mechanisms of dormancy and early stages of metastasis, which are conceptually decisive in the seeding of bone by tumor cells. These cells become resistant to treatments and, after a period of seemingly absent disease, return to take part in a devastating vicious cycle.

Accepted: May 5, 2021
